# In Vivo Wear Analysis of Leucite-Reinforced Ceramic Inlays/Onlays After 14 Years

**DOI:** 10.3390/ma18153446

**Published:** 2025-07-23

**Authors:** Ragai-Edward Matta, Lara Berger, Oleksandr Sednyev, Dennis Bäuerle, Eva Maier, Werner Adler, Michael Taschner

**Affiliations:** 1 Department of Prosthodontics, Erlangen University Hospital, Glueckstrasse 11, 91054 Erlangen, Germany; lara.berger@uk-erlangen.de (L.B.); oleksandr.sednyev@uk-erlangen.de (O.S.); 2 Department of Operative Dentistry and Periodontology, Erlangen University Hospital, Glueckstrasse 11, 91054 Erlangen, Germany; baeuerle.dennis@hotmail.de (D.B.); eva.maier@uk-erlangen.de (E.M.); michael.taschner@uk-erlangen.de (M.T.); 3 Department of Medical Informatics, Biometry and Epidemiology, Friedrich-Alexander-University of Erlangen-Nuremberg, Waldstrasse 6, 91054 Erlangen, Germany; werner.adler@uk-erlangen.de

**Keywords:** occlusal interactions, wear of glass-ceramic, wear behavior, all-ceramic restorations, ceramic inlays/onlays, long-term clinical investigation

## Abstract

Material wear significantly impacts the clinical success and longevity of dental ceramic restorations. This in vivo study aimed to assess the wear behavior of IPS Empress^®^ glass-ceramic inlays and onlays over 14 years, considering the influence of different antagonist materials. Fifty-four indirect restorations of 21 patients were available for comprehensive wear analysis, with complete follow-up data for up to 14 years. Three-dimensional measurements relied on digitized epoxy resin models produced immediately post-insertion (baseline) and subsequently at 2, 4, and 14 years. The occlusal region on the baseline model was delineated for comparative analysis. Three-dimensional superimpositions with models from subsequent time points were executed to assess wear in terms of average linear wear and volumetric loss. Statistical analyses were conducted in R (version 4.4.1), employing Mann–Whitney U tests (material comparisons) and Wilcoxon signed rank tests (time point comparisons), with a significance threshold of *p* ≤ 0.05. During the entire study period, an increase in wear was observed at each assessment interval, gradually stabilizing over time. Significant differences in substance loss were found between the follow-up time points, both for mean (−0.536 ± 0.249 mm after 14a) and integrated distance (−18,935 ± 11,711 mm^3^ after 14a). In addition, significantly higher wear was observed after 14 years with gold as antagonist compared to other materials (*p* ≤ 0.03). The wear behavior of IPS Empress^®^ ceramics demonstrates clinically acceptable long-term outcomes, with abrasion characteristics exhibiting stabilization over time.

## 1. Introduction

Indirect single-tooth ceramic restorations, such as inlays and onlays, offer advantages over direct composite restorations, when restoring larger defects [1,2,3]. These techniques have been shown to present good long-term survival rates and have proven to be reliable in everyday clinical practice [4,5].

In general, modern dental ceramics are known for their excellent esthetic properties, biocompatibility, and durability [6,7]. Glass-ceramics have color properties similar to human enamel and are particularly translucent due to their high glass content; although, this can lead to an impairment of mechanical properties. These mechanical limitations are partially compensated by an increased crystalline content [8].

With the market launch of IPS Empress^®^ from Ivoclar Vivadent AG (Schaan, Lichtenstein), an attempt was made to optimize mechanical properties by stabilizing the glass matrix through integration of leucite crystals and thus to develop a highly esthetic yet stable restorative material [9]. In this context, a review on longevity and clinical performance showed a survival rate of up to 91% for IPS Empress^®^ inlays and onlays after seven years, which underlines the reliability and durability of this material [10].

However, long-term success is limited by factors such as secondary caries, adhesive failure, and fractures [11]. In addition, the experience of the dentist and the correct indication for the restoration of lost dental hard tissue are decisive influencing parameters for long-term outcomes [12].

In this respect, wear mechanisms are determined by a variety of influencing variables, including individual masticatory forces, the shape and size of tooth contact surfaces, and the surface texture, microstructure, and fracture strength of the material [13]. The hardness of the material proves to be less decisive, while the surface roughness and fracture toughness of the ceramic are of greater importance [14]. In principle, the occlusal wear properties of the antagonists should be comparable to ensure long-term functionality without structural deficits [15,16,17].

Concerning tribology, various primary mechanisms are described with regard to wear processes. These include chemical dissolutive transformations, in which material is removed by chemical reactions, as well as adhesive wear, which is caused by adhering material particles that are subsequently torn off at the contact surfaces. Other parameters include surface fatigue, which occurs when repeated stresses lead to microcracks and consequential failure of the material, or mechanical abrasion, when material is worn away by particles of variable hardness [18,19]. In this context, patient-specific factors, such as bruxism, forced tooth brushing, diet, or salivary flow, also play a central role [13,20,21]. Various systems are available for tribological investigations, with both two- and three-body wear testing systems frequently used to analyze the wear resistance of materials under realistic conditions [18,22,23]. To evaluate the results of these laboratory testing approaches, industrial light–optical scanners enable contactless and highly precise object registration in form of three-dimensional (3D) datasets, which can then be virtually aligned with each other and precisely superimposed or aligned using special software [24,25,26].

According to an in vitro study, leucite-reinforced glass-ceramics exhibit improved abrasion resistance [27]. Zirconia ceramics are characterized by lower wear rates compared to lithium disilicate ceramics, which consequently results in reduced material loss [28]. Compared to lithium disilicate ceramics, gold restorations show a higher abrasiveness in vitro, especially with IPS Empress^®^ ceramics [29]. While there is a vast body of work regarding in vitro wear behavior of IPS Empress^®^ [28], recent literature lacks conclusive evidence addressing the abrasion behavior of dental ceramics against various antagonist, particularly IPS Empress^®^ in clinical context [30]. Therefore, laboratory studies conducted under defined and controlled conditions cannot simulate all the individual and complex factors of the chewing process [16,31]. This implies the need for long-term clinical studies to record the wear behavior under real conditions and thus evaluate the actual performance of different materials.

The aim of this in vivo study was, therefore, to quantitatively determine the occlusal wear rates of IPS Empress^®^ glass-ceramic inlays and onlays in available patients over follow-up intervals of two, four, and fourteen years correlated with the respective antagonist material in order to gain a comprehensive understanding of the long-term effects and clinical performance of this material.

## 2. Materials and Methods

### 2.1. Study Design

This prospective clinical study aimed to evaluate the wear behavior of leucite-reinforced glass-ceramic restorations using a light–optical 3D scanning technique by digitally comparing virtual patient models of the intraoral situations after 2, 4, and a total observation period of 14 years. The study project was approved in advance by the Ethics Committee of the University Hospital Erlangen (No. 2628) and conducted in accordance with EN 540 (Clinical investigation of medical devices in humans, European Committee for Standardization), the CONSORT declaration [32], and established protocols [33,34].

### 2.2. Subject Recruitment and Intervention

An experienced assistant professor treated 30 selected patients (average age 39.4 years; age ranging between 23–64 years; eleven men, nineteen women) at the Department of Conservative Dentistry and Periodontology at the University Hospital Erlangen according to defined inclusion criteria (Table 1 [35,36]). All participants gave their written informed consent, agreed to the study procedure, and committed to regular check-ups in the first 4 years after placement of the restoration; participation in the 14-year check-up was voluntary.

At the beginning of the study, a total of 83 IPS Empress^®^ restorations were placed in 30 patients, consisting of 31 two-surface inlays, 39 MOD (mesial-occlusal-distal) inlays, and 13 onlays, which were distributed over 34 upper premolars, 14 upper molars, 13 lower premolars, and 22 lower molars.

Cavity preparations followed standardized protocols to ensure consistent quality and geometry, as described in previous publications [35,36]. Cavities close to the pulp were excluded to avoid adhesion reduction by calcium hydroxide. After preparation, two-phase polyvinylsiloxane impressions were taken (Dimension Garant H and Garant L, 3M, Seefeld, Germany), and chairside-fabricated temporary restorations (Luxatemp, DMG, Hamburg, Germany) were fixed with TempBond^TM^ NE (Kerr, Herzogenrath, Germany). The leucite-reinforced glass-ceramic restorations were produced in a commercial dental laboratory according to manufacturer’s instructions [37].

To ensure that the maximum cement gap width of 100 μm was not exceeded, a special dental probe was used (tip diameter 100 µm, tactile control). Before insertion, the thickness of the restorations was measured with a caliper (rapid caliper, accuracy 0.01 mm, Kroeplin, Schluechtern, Germany) to check a sufficient minimum layer thickness at the relevant points. Afterwards, the inlays and onlays were adhesively bonded according to the clinical instructions for the pre-treatment of tooth and restoration [36]. After removing excess insertion material and the rubber dam, occlusal contacts were adjusted with fine diamond drills. Final polishing was performed with diamond and polishing strips interdentally (GC Dental Industrial Corp., Tokyo, Japan/3M), while the occlusal finish was achieved using felt discs (Dia-Finish E felt discs, Renfert, Hilzingen, Germany) and diamond polishing paste (Brinell, Renfert).

**Figure 1:**
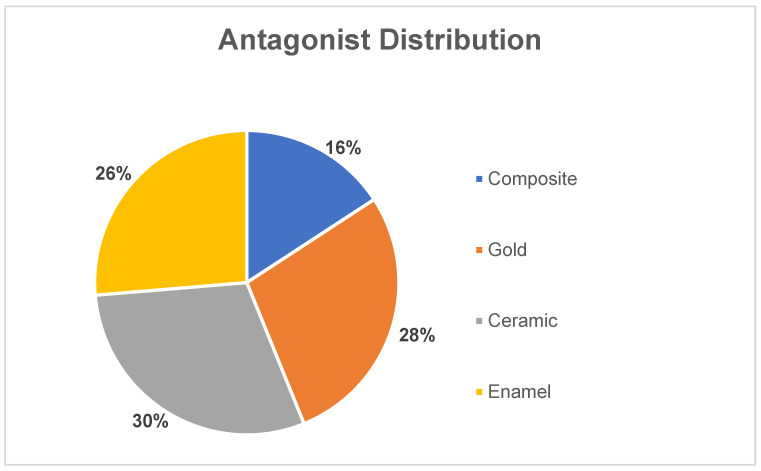
Pie chart of the antagonist distribution in percentage.

The follow-up examinations of the restorations were carried out at the following four points in time: after 2 weeks (=baseline) and after 2, 4 and 14 years. Double-mix impressions (Panasil, Kettenbach GmbH und Co. KG, Eschenburg, Germany) were taken, subsequently filled with epoxy resin (AlphaDie MF, Schütz Dental GmbH, Rosbach, Germany) and stored as precision models in the Dental Clinic 1—Conservative Dentistry and Periodontology—of the University Hospital Erlangen, Germany. In addition, the respective antagonists (enamel, composite, ceramic, gold) were recorded by means of photography (Figure 1).

### 2.3. Three-Dimensional Data Acquisition and Volumetric Measurements

In preparation for the subsequent digitization processes, all physical patient models were equipped with ATOS 0.8 mm reference points (GOM GmbH, Braunschweig, Germany) to enable spatial orientation and thus linking areas of the individual measurements to a 3D overall representation of the object. In view of the subsequent evaluations, these were only attached to non-relevant areas, i.e., not to restorations and not to areas necessary for the virtual superimposition process. Furthermore, to improve the scan quality, matting was carried out using a titanium dioxide solution applied with an airbrush gun (RICH^®^ AS-2, Fuso Seiki Co., Ltd., Tokyo, Japan), of which the minimum layer thickness can be considered negligible concerning measurements [38,39].

An industrial, high-precision non-contact scanner (ATOS So4 II, GOM GmbH) was used to digitize the models, as it has a smaller measurement error of less than 0.004 mm in the course of object registration compared to conventional dental laboratory scanners for the measurement volume used here [40]. The ATOS So4 II system was calibrated at the beginning of each measurement sessions in accordance with the manufacturer’s instructions by a specifically manufacturer-trained employee of our faculty. All measurements were performed by a trained and experienced user. Based on the triangulation principle, precise light points were projected onto the object to be measured, reflected, and recorded by two light-sensitive sensors arranged at defined angles [41]. Several individual measurements per patient model from different perspectives resulted in a measurement series of point clouds, which could be transformed into a common coordinate system using the reference points (GOM GmbH) attached in advance. In this way, four precise virtual 3D models were generated for each patient case in the common.stl (Surface/Standard Triangulation/Tesselation Language) format. Afterwards, the scanner’s analysis software (GOM Inspect Professional 2018, GOM GmbH) was used to perform a digital data analysis by comparing (=matching) the occlusal surfaces of the restorations to be examined (=region of interest, ROI) between the models of the follow-up examinations (after 2, 4 and 14 years; =IST models) and the baseline model (after 2 weeks; =SOLL model) in order to evaluate the occlusal material loss of the restorations both numerically and graphically. In the course of the respective target–actual superimposition for the before-and-after comparisons at different time points, the model pairs were first roughly aligned manually by selecting prominent points in the central fissures or on the cusp tips. In a second step, the surfaces of the teeth adjacent to the restorations were selected, and an automated fine alignment was performed using a “best fit” algorithm.

**Figure 2:**
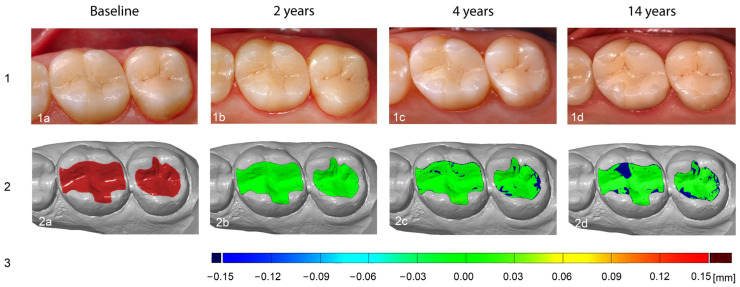
Clinical and virtual 3D visualization of occlusal wear over a 14-year period. Series (**1a**–**1d**): Clinical situations in an occlusal view. Series (**2**): Corresponding 3D surface models. (**2a**): Baseline model with defined region of interest (ROI). (**2b**–**2d**): Superimpositions of follow-up models with baseline. Color-coded distance maps illustrate material loss, with blue denoting wear and green indicating areas with no or almost no surface loss. Series (**3**): Color-coded scale bar to quantify surface changes.

This standardized procedure ultimately enabled highly precise recording of the occlusal volume changes in the form of color-coded distance maps to visualize local differences due to wear processes (Figure 2). In addition, a numerical calculation of the average surface loss (“mean distance” in [mm]) and the volume decrease (“integrated distance” in [mm^3^]) over time was carried out.

### 2.4. Statistical Data Analysis

**Figure 3:**
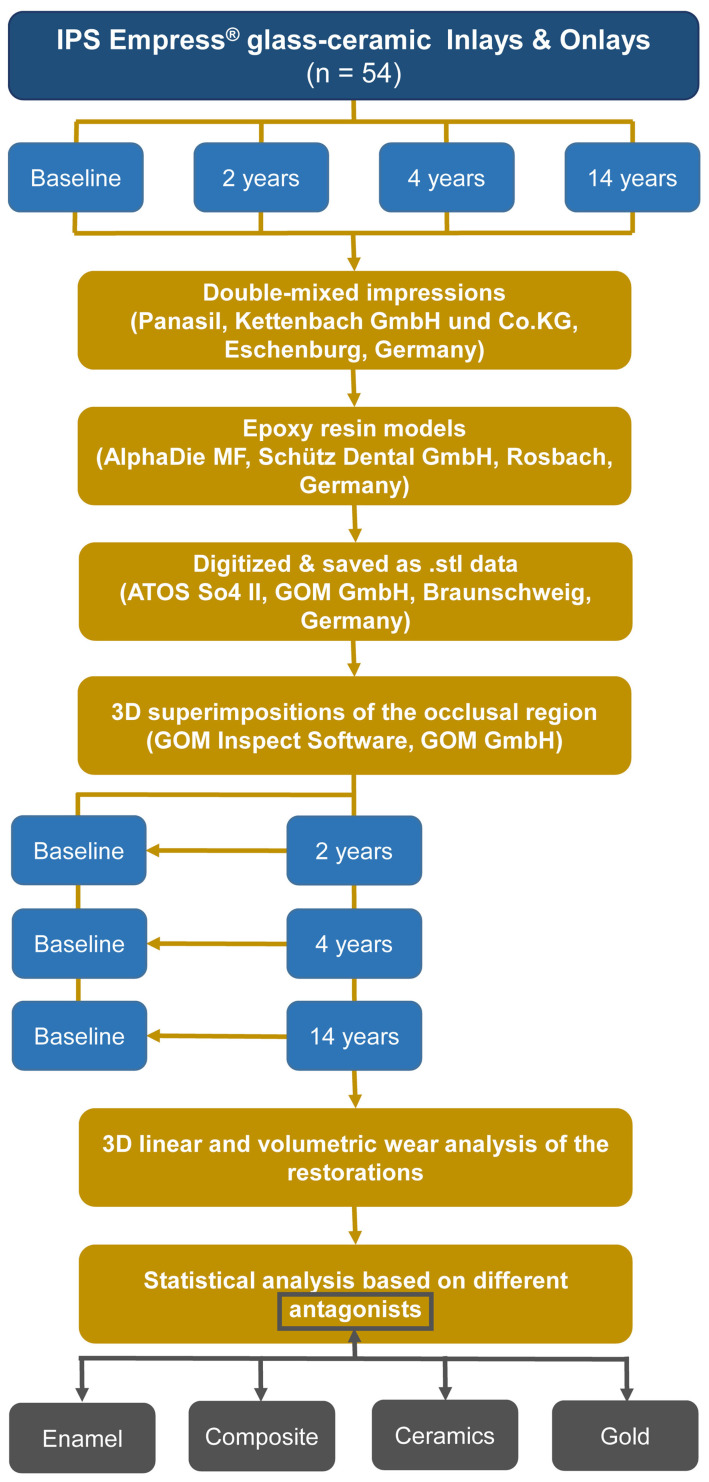
Flowchart illustrating the study design.

Statistical analyses to evaluate the wear on the IPS Empress^®^ restorations in relation to the respective antagonist class were conducted using the R programming language (version 4.4.1) [42]. Mann–Whitney U tests (comparing different materials at the same time point) and Wilcoxon signed rank tests (comparing different time points within one material) were used to evaluate the data. The significance level was set at *p* ≤ 0.05. An overview of the study procedure is shown in Figure 3.

## 3. Results

### 3.1. Study Population

The investigations were based on digital patient models at the various follow-up times, with a total number of 83 IPS Empress^®^ inlays/onlays (baseline, n = 83) in 30 patients at the beginning. Over time, the number decreased so that after 2 years, a total of 82 restorations (98.8%); after 4 years, 72 inlays and onlays (86.75%); and after a period of 14 years, 54 IPS Empress^®^ glass-ceramic restorations (65%) were still included in the study. The reasons were failure (16.9%), change in residence, or death (18.1%), with a failure rate of 12% according to the Kaplan–Meier analysis [36]. During the last follow-up examination, the sample consisted of 21 patients, including 13 women and 8 men aged between 24 and 66 years (mean: 40 years).

### 3.2. Substance Loss Analysis Methodology

The results for the evaluation of the occlusal wear rates were derived exclusively from negative measured values up to a maximum limit of 0.00 mm, which in turn would not correspond to any substance loss. These negative values illustrate the extent of height loss of the restoration as a result of the effect of the corresponding antagonists.

### 3.3. Average Surface Loss

**Figure 4:**
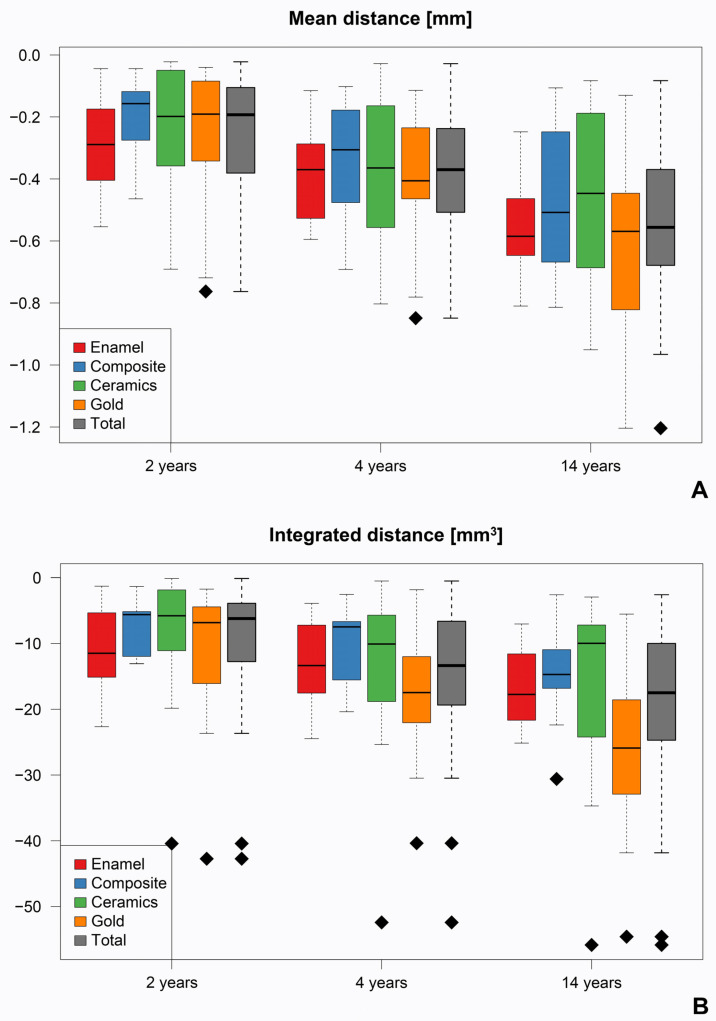
Boxplot diagrams depicting the occlusal wear of the ceramic restoration in terms of average surface loss (mean distance) (**A**) and volume decrease (integrate distance) (**B**) after 2, 4, and 14 years. The black rhombus symbols indicate outliers.

Over the course of the entire study period, the average surface loss of the IPS Empress^®^ glass-ceramic inlays and onlays showed a progressive increase, which occurred independently of the antagonist material present. Two years after placement of the restorations, there was a total loss of −0.253 ± 0.185 mm; after four years, −0.380 ± 0.205 mm; and after fourteen years, −0.536 ± 0.249 mm. The comparison of the different follow-up times (2 vs. 4 years/2 vs. 14 years/4 vs. 14 years) within the same antagonist group (enamel, composite, ceramic, gold) provided consistently significant results (*p* ≤ 0.004). In contrast, the comparison of the different antagonists with regard to abrasion on the ceramic restorations showed no significant differences after 2, 4, and 14 years (Figure 4, Table 2).

### 3.4. Volume Decrease

The occlusal volume of the inlays and onlays also decreased over time, resulting in a total volume loss of −9981 ± 8965 mm^3^ after 2 years, −14,207 ± 9753 mm^3^ after 4 years, and −18,935 ± 11,711 mm^3^ after 14 years. The comparative analysis of the integrated distance between the time points 2 and 4 years, 2 and 14 years, and 4 and 14 years after final placement of the ceramic restorations showed consistently significant results for all antagonists, analogous to the average surface loss (*p* ≤ 0.02). Furthermore, a significantly higher material loss was determined after 14 years if gold was present as an antagonist (*p* < 0.03) (Figure 4, Table 2).

## 4. Discussion

In this study, a 3D surface analysis was conducted to evaluate the abrasive properties of 54 IPS Empress^®^ restorations over an observation period of 14 years in relation to different antagonists. In this respect, long-term clinical studies are essential in order to be able to adequately assess the suitability of dental ceramics for long-term use in patients and thus for clinical success [43]. The physical properties of the ceramic are of particular importance in order to withstand abrasive challenges in the oral cavity [44] and ultimately ensure satisfactory long-term performance for both the patient and the practitioner [10].

Many of the studies on abrasion behavior of leucite-reinforced glass-ceramic inlays found in the literature are in vitro studies that evaluate the wear of the antagonistic tooth and not of the restoration itself [45,46]. However, the quantitative comparison of in vitro and in vivo results shows a high correlation of the data and the associated relevance of laboratory studies for clinical performance [47]. Nevertheless, clinical studies are considered the most valuable method for determining wear behavior [17,48].

In the present analysis, the abrasion on the restorations of 13 female and 8 male test subjects aged 24 to 66 years was analyzed over the entire observation period and thus with a heterogeneous gender and age distribution. In this regard, Ohlmann et al. [49] showed in their study investigating the abrasive behavior of posterior crowns made of different materials using 3D laser scanning technology that neither the age nor the gender of the patient collective had a significant influence on wear.

Overall, the results obtained show that a continuous and significant increase in surface and volume loss occurred in IPS Empress^®^ glass-ceramic inlays and onlays up to 14-years in vivo, irrespective of the antagonist material used. The comparison of the measured values for abrasion in the occlusal contact area between the individual examination intervals showed significant differences for both the mean distance and integrated distance compared to the individual antagonist groups, with the abrasion rate being more pronounced in the first few years and then decreasing (*p* ≤ 0.02). Over the course of the 2-, 4-, and 14-year follow-up periods, no statistically significant differences in restoration wear were observed between the various antagonist materials, with the exception of a significantly greater volumetric material loss associated with gold antagonists at the 14-year recall (*p* < 0.03) (Figure 2, Table 2). These findings indicate that composite, ceramic, and enamel antagonists exhibit comparable long-term wear behavior when opposing IPS Empress^®^ restorations. Accordingly, both ceramic and composite antagonists may be regarded as suitable and clinically favorable counterparts for IPS Empress^®^ restorations in long-term applications. In view of the fact that natural and healthy enamel is subject to a continuous annual abrasion process of around 20 to 40 μm in the molar region even under physiological conditions [13,15], the loss of material on the occlusal surfaces of the restorations observed in this study after just two years can be considered appropriate. The FDI clinical criteria specify that restorations exhibiting wear within 80–120% of the enamel wear are deemed harmonious, while wear substantially exceeding the natural enamel wear—exceeding 300 µm occlusally—is regarded as problematic [50]. Thus, if a restoration’s wear mimics enamel wear in both its rate and pattern, it is considered clinically acceptable even if some volume loss or subtle changes in contour become apparent over time. The fact that abrasion processes occur on ceramic restorations after a short time has already been confirmed in vivo by Aladağ et al. [48] using 3D data overlays. An average volumetric abrasion of 0.27 ± 0.16 mm^3^ was evaluated on lithium disilicate molar crowns after only six months. However, it must be noted that the hardness of a material is not the only determining factor for wear. The masticatory loads and the associated wear processes in the oral cavity represent a complex, multifactorial phenomenon that is influenced by numerous individual factors [51]. In the study by Aladağ et al. [48], patient-specific factors that influence the complex intraoral abrasion process and the associated material wear [13,15] were not documented, as is the case in the present study, which should be seen as a limitation. The variability of these factors, such as chewing pattern, saliva composition, and individual dietary habits, could affect the interpretation and transferability of the results. However, the relationship between maximum biting force and the wear rates of enamel and all-ceramic crowns, for example, was investigated in a clinical study, but no significant correlation was found [51]. Furthermore, other authors point out that the additional recording of food consumption over such a long observation period, which in the present study would cover more than 10% of a person’s average lifespan, is not considered realistic [34].

Krejci et al. [52] analyzed the abrasion of MOD inlays made of four different ceramic materials on a total of 24 extracted, caries-free molars using chewing simulation. Palatal cusps of human maxillary first molars of similar shape and dimensions, which showed no existing abrasion, were used as antagonists. The investigations were carried out in a computer-controlled chewing simulator with six chambers, which simulated real chewing processes and in vivo load over five years. The samples were subjected to several factors, including chemical degradation (75% ethanol at 37 °C), mechanical toothbrush abrasion (2 N), cyclic masticatory loads (max. 49 N at 1.7 Hz), and thermal cycles in water with alternating temperatures of 5 °C and 55 °C. A material loss of −21.8 ± 8.8 µm was measured in the occlusal contact point area for polished IPS Empress^®^ restorations. In contrast, significantly higher values in the tenths of a millimeter range were recorded in the present study after only two years. This can be explained by the fact that the present study was conducted under real in vivo conditions, whereas Krejci et al. [52] conducted their study in vitro with only six test objects per group and enamel as the only antagonist, which limits the comparability of the results. In addition, not only molars but also premolars were included in the analyses in the current study. However, it has already been shown that there is no direct correlation between the loss of substance and the specific location of the inlays in the oral cavity [34].

In contrast, Krämer et al. [34] conducted a clinical study to evaluate the wear phenomena of 17 ceramic inlays on premolars and molars made of IPS Empress^®^ against enamel antagonists over a period of 8 years. The average occlusal wear of the ceramic inlays analyzed using the 3D evaluation method was 78 µm after 4 years and 116 µm after 8 years, with the difference between the two time points being statistically significant. While the measured values are lower compared to the results of the present study, but tend to be comparable, the data of the current analysis also show significant differences between the examination intervals. This indicates progressive wear over time; although, this appears to be stabilizing.

In this regard, Lambrechts et al. [15] reported a tendency towards higher wear rates in the first few years (initial phase), which subsequently stabilize as the occlusal environment approaches a dynamic equilibrium (steady-state phase), provided no further restorative measures are taken. Consequently, the wear process shows a non-linear progression, as the initially small contact areas can lead to high pressure concentrations, which in turn cause an increased wear rate [52,53]. This is consistent with the results of the study conducted, as after only 2 years, depending on the antagonist group, around 40–60% of the total material loss observed after 14 years occurred.

In addition, significantly higher wear was observed on the ceramic restorations after 14 years when gold was used as an antagonist (*p* ≤ 0.03). This contrasts with the observations of a different in vitro study, which showed that gold in particular, and less so ceramic, exhibited accelerated wear when in contact with each other. However, the authors also describe a “functional polishing” that occurs on ceramic surfaces at the beginning of a masticatory load and removes the rough surface layers. Thus, progressive polishing of the ceramic leads to a gradual decrease in the abrasion rate. However, when gold was present as an antagonist, little evidence of these polishing mechanisms of the ceramic surface was documented, and instead, rapid wear of the gold was observed. Furthermore, gold particles were observed to transfer to the ceramic surfaces over time [53]. These microparticles could act as abrasives and thus lead to increased abrasion processes, as the surface roughness is directly related to the abrasion rate [29,53,54].

In contrast to the gold antagonists, the IPS Empress^®^ inlays and onlays showed no significant differences in abrasion behavior compared to composite, ceramic, and natural enamel at the individual follow-up times. This indicates a comparable wear resistance and confirms the compatibility of these material classes (with the exception of gold) for long-term clinical use.

Overall, there are only a few in vivo studies in the literature that prospectively document the vertical dimensional loss of ceramic restorations, particularly inlays and onlays, over a longer period of time [34,55], which limits the comprehensive comparability of the results. The focus of this analysis was on the abrasion of ceramic restorations as a function of different antagonists, which has not yet been investigated in this form.

Although mechanical profilometry was previously described as a reliable method for determining surface changes [56], the present study is based on a modern digital 3D laser scanning method (ATOS So4 II, GOM GmbH) of replica models. The choice of measurement method significantly influences the accuracy and reliability of the results [17,57]. The ATOS system used generates high-precision and non-contact digitally recorded measurement data that can be analyzed quantitatively and qualitatively reproducibly in both two- and three-dimensional form [58]. In general, the use of 3D measurement techniques for the visualization of surface changes has become established in scientific research, as the smallest changes can be recorded with the highest precision and attention to detail [17,34,48,51]. Double-mix impressions, which were taken at each examination time and poured with epoxy resin, served as the basis for the measurements carried out. Both materials have been shown to be characterized by high detail accuracy, dimensional stability, and storage stability, thus enabling the precise reproduction of the intraoral structures [59,60]. Alternatively, plaster could have been used for model fabrication. However, the detail reproduction would have been limited to 20 microns with Class IV plaster compared to epoxy resin, which reveals structural details down to one micron [60]. Another option would have been digital impression taking using intraoral scanning, which has been shown to offer comparable accuracy to conventional methods and eliminates the need for physical model fabrication but was not available at the beginning of the present study. However, data capture is limited to visible areas, which can be a challenge in hard-to-reach regions. In addition, blood, saliva, and other patient-specific factors can affect image quality, while precision may be lower than with conventional techniques in large dental arches [61]. Furthermore, a recent in vitro study showed that an industrial precision scanner from GOM GmbH generates the highest precision in object registration compared to laboratory scanners, photogrammetry devices, and intraoral scanners [62].

Overall, it should be mentioned that the analysis regarding the effect of different antagonist materials was not the focus of the study design when the present investigation was initiated, hence a control group is missing. At that point in time, the influence of different antagonists on wear of restorations was not yet a focus in the scientific literature. The lack of patient-specific factors and the comparably small sample size present further limitations within the scope of this study. Nevertheless, our data reflect real clinical conditions, in which a large number of different antagonist materials naturally occur.

Since the wear of dental materials is subject to numerous influencing factors and mechanisms, further data from long-term clinical studies are essential for a sound understanding of the relationships between wear rate and relevant parameters [47]. Future research should use a standardized method to investigate wear, such as the 3D analysis used in this study, in order to improve the comparability of the data to the already complex mechanisms of material wear. In addition, research should be conducted into potential selection tools based on artificial intelligence (AI) that take antagonists into account.

## 5. Conclusions

Conclusively, the evaluated results indicate that IPS Empress^®^ ceramic restorations exhibit adequate wear resistance according to the FDI, which is stabilized due to superficial polishing processes, and can, therefore, be recommended for long-term clinical use.

Concerning the long-term success of restorative materials, such as the glass-ceramic inlays and onlays examined in this study, it is important to consider comparable wear properties of the opposing antagonists, to ensure the long-term integrity and functionality of dental restorations.

## Figures and Tables

**Table 1:** Conditions of the study population.

Inclusion Criteria
- Symptom-free tooth for restoration - Rubber dam application possible - No further treatment needed for other posterior teeth - Good oral hygiene - No periodontal or pulpal disease - Age 18–65 - No deep cavities in close proximity to the pulp

**Table 2:** *p*-values for comparisons of average surface loss (mean distance) and volume decrease (integrated distance) depending on different antagonists at one point in time and between different points in time within an antagonist material.

Mean Distance [mm]			
Material comparison	2 years	4 years	14 years
Enamel vs. Composite	0.27	0.421	0.743
Enamel vs. Ceramic	0.216	0.767	0.333
Enamel vs. Gold	0.412	1.000	0.709
Composite vs. Ceramic	0.955	0.777	0.777
Composite vs. Gold	0.858	0.421	0.411
Ceramic vs. Gold	0.752	0.922	0.202
Time point comparison	2 vs. 4 years	2 vs. 14 years	4 vs. 14 years
Enamel	<0.001	<0.001	0.001
Composite	0.004	0.004	0.004
Ceramic	<0.001	<0.001	<0.001
Gold	<0.001	<0.001	<0.001
Integrated Distance [mm^3^]			
Material comparison	2 years	4 years	14 years
Enamel vs. Composite	0.194	0.411	0.421
Enamel vs. Ceramic	0.163	0.626	0.358
Enamel vs. Gold	0.806	0.233	0.026
Composite vs. Ceramic	0.978	0.760	0.760
Composite vs. Gold	0.482	0.108	0.025
Ceramic vs. Gold	0.304	0.140	0.030
Time point comparison	2 vs. 4 years	2 vs. 14 years	4 vs. 14 years
Enamel	0.002	<0.001	0.004
Composite	0.004	0.004	0.020
Ceramic	<0.001	<0.001	0.005
Gold	<0.001	<0.001	0.003

## Data Availability

The raw data supporting the conclusions of this article will be made available by the authors on request.

## Abbreviations

The following abbreviations are used in this manuscript:

3D	three-dimensional
EN	European norm
MOD	Mesial–occlusal–distal
ROI	region of interest
CONSORT	Consolidated Standards of Reporting Trials

## References

[1] Meyer A., Cardoso L.C., Araujo E., Baratieri L.N. (2003). Ceramic inlays and onlays: Clinical procedures for predictable results. J. Esthet. Restor. Dent..

[2] Hopp C.D., Land M.F. (2013). Considerations for ceramic inlays in posterior teeth: A review. Clin. Cosmet. Investig. Dent..

[3] Al-Fouzan A.F., Tashkandi E.A. (2013). Volumetric measurements of removed tooth structure associated with various preparation designs. Int. J. Prosthodont..

[4] Collares K., Corrêa M.B., Laske M., Kramer E., Reiss B., Moraes R.R., Huysmans M.C., Opdam N.J. (2016). A practice-based research network on the survival of ceramic inlay/onlay restorations. Dent. Mater..

[5] Forna N.C., Uleanu R., Lungu I., Dimofte A.-R. (2024). Dental ceramics: Advantages and benefits. Practice.

[6] Contrepois M., Soenen A., Bartala M., Laviole O. (2013). Marginal adaptation of ceramic crowns: A systematic review. J. Prosthet. Dent..

[7] Kelly J.R., Benetti P. (2011). Ceramic materials in dentistry: Historical evolution and current practice. Aust. Dent. J..

[8] Sen N., Us Y.O. (2018). Mechanical and optical properties of monolithic CAD-CAM restorative materials. J. Prosthet. Dent..

[9] Albakry M., Guazzato M., Swain M.V. (2004). Influence of hot pressing on the microstructure and fracture toughness of two pressable dental glass-ceramics. J. Biomed. Mater. Res. B Appl. Biomater..

[10] El-Mowafy O., Brochu J.F. (2002). Longevity and clinical performance of IPS-Empress ceramic restorations—A literature review. J. Can. Dent. Assoc..

[11] Abdulrahman S., Von See Mahm C., Talabani R., Abdulateef D. (2021). Evaluation of the clinical success of four different types of lithium disilicate ceramic restorations: A retrospective study. BMC Oral Health.

[12] Manhart J., Chen H., Hamm G., Hickel R. (2004). Buonocore Memorial Lecture. Review of the clinical survival of direct and indirect restorations in posterior teeth of the permanent dentition. Oper. Dent..

[13] Yip K.H., Smales R.J., Kaidonis J.A. (2004). Differential wear of teeth and restorative materials: Clinical implications. Int. J. Prosthodont..

[14] Sripetchdanond J., Leevailoj C. (2014). Wear of human enamel opposing monolithic zirconia, glass ceramic, and composite resin: An in vitro study. J. Prosthet. Dent..

[15] Lambrechts P., Braem M., Vuylsteke-Wauters M., Vanherle G. (1989). Quantitative in vivo wear of human enamel. J. Dent. Res..

[16] Dupriez N.D., von Koeckritz A.K., Kunzelmann K.H. (2015). A comparative study of sliding wear of nonmetallic dental restorative materials with emphasis on micromechanical wear mechanisms. J. Biomed. Mater. Res. B Appl. Biomater..

[17] de Carvalho Ramos N., Augusto M.G., Alves L.M.M., Kleverlaan C.J., Dal Piva A.M.d.O. (2023). Wear of dental ceramics. Braz. Dent. Sci..

[18] Osiewicz M.A., Werner A., Roeters F.J.M., Kleverlaan C.J. (2019). Wear of direct resin composites and teeth: Considerations for oral rehabilitation. Eur. J. Oral Sci..

[19] Schlueter N., Amaechi B.T., Bartlett D., Buzalaf M.A.R., Carvalho T.S., Ganss C., Hara A.T., Huysmans M., Lussi A., Moazzez R. (2020). Terminology of Erosive Tooth Wear: Consensus Report of a Workshop Organized by the ORCA and the Cariology Research Group of the IADR. Caries Res..

[20] Lobbezoo F., Ahlberg J., Raphael K.G., Wetselaar P., Glaros A.G., Kato T., Santiago V., Winocur E., De Laat A., De Leeuw R. (2018). International consensus on the assessment of bruxism: Report of a work in progress. J. Oral Rehabil..

[21] Dahl B.L., Carlsson G.E., Ekfeldt A. (1993). Occlusal wear of teeth and restorative materials. A review of classification, etiology, mechanisms of wear, and some aspects of restorative procedures. Acta Odontol. Scand..

[22] Maier E., Grottschreiber C., Knepper I., Opdam N., Petschelt A., Loomans B., Lohbauer U. (2022). Evaluation of wear behavior of dental restorative materials against zirconia in vitro. Dent. Mater..

[23] Someya T., Kasahara M., Takemoto S., Hattori M. (2023). The Wear Behavior of Glass-Ceramic CAD/CAM Blocks against Bovine Enamel. Materials.

[24] Holst S., Karl M., Wichmann M., Matta R.E. (2012). A technique for in vitro fit assessment of multi-unit screw-retained implant restorations: Application of a triple-scan protocol. J. Dent. Biomech..

[25] Jeon J.H., Kim H.Y., Kim J.H., Kim W.C. (2014). Accuracy of 3D white light scanning of abutment teeth impressions: Evaluation of trueness and precision. J. Adv. Prosthodont..

[26] Jeon J.H., Choi B.Y., Kim C.M., Kim J.H., Kim H.Y., Kim W.C. (2015). Three-dimensional evaluation of the repeatability of scanned conventional impressions of prepared teeth generated with white- and blue-light scanners. J. Prosthet. Dent..

[27] Theocharopoulos A., Chen X., Hill R., Cattell M.J. (2013). Reduced wear of enamel with novel fine and nano-scale leucite glass-ceramics. J. Dent..

[28] Cherian J., Jayakumar R., James J., Thomas V., Sramadathil S., Sasi A.K. (2023). A comparative evaluation of enamel wear against different surface finished ceramics: An in vitro study. Cureus.

[29] Elmaria A., Goldstein G., Vijayaraghavan T., Legeros R.Z., Hittelman E.L. (2006). An evaluation of wear when enamel is opposed by various ceramic materials and gold. J. Prosthet. Dent..

[30] Etman M. (2013). Wear properties of dental ceramics. Non-Metallic Biomaterials for Tooth Repair and Replacement.

[31] Heintze S.D., Cavalleri A., Forjanic M., Zellweger G., Rousson V. (2008). Wear of ceramic and antagonist--a systematic evaluation of influencing factors in vitro. Dent. Mater..

[32] Needleman I., Worthington H., Moher D., Schulz K., Altman D.G. (2008). Improving the completeness and transparency of reports of randomized trials in oral health: The CONSORT statement. Am. J. Dent..

[33] Frankenberger R., Taschner M., Garcia-Godoy F., Petschelt A., Krämer N. (2008). Leucite-reinforced glass ceramic inlays and onlays after 12 years. J. Adhes. Dent..

[34] Krämer N., Kunzelmann K.-H., Taschner M., Mehl A., Garcia-Godoy F., Frankenberger R. (2006). Antagonist enamel wears more than ceramic inlays. J. Dent. Res..

[35] Taschner M., Frankenberger R., Garcia-Godoy F. (2009). IPS Empress inlays luted with a self-adhesive resin cement after 1 year. Am. J. Dent..

[36] Taschner M., Stirnweiss A., Frankenberger R., Kramer N., Galler K.M., Maier E. (2022). Fourteen years clinical evaluation of leucite-reinforced ceramic inlays luted using two different adhesion strategies. J. Dent..

[37] Beham G. (1991). IPS Empress: Eine Neue Keramik Techologie.

[38] Vlaar S.T., van der Zel J.M. (2006). Accuracy of dental digitizers. Int. Dent. J..

[39] Matta R.E., von Wilmowsky C., Neuhuber W., Lell M., Neukam F.W., Adler W., Wichmann M., Bergauer B. (2016). The impact of different cone beam computed tomography and multi-slice computed tomography scan parameters on virtual three-dimensional model accuracy using a highly precise ex vivo evaluation method. J. Craniomaxillofac. Surg..

[40] Holst S., Karl M., Wichmann M., Matta R.E. (2011). A new triple-scan protocol for 3D fit assessment of dental restorations. Quintessence Int..

[41] Zimmermann M., Mehl A., Mörmann W.H., Reich S. (2015). Intraoral scanning systems—A current overview. Int. J. Comput. Dent..

[42] R Core Team (2024). R: A Language and Environment for Statistical Computing.

[43] Malament K.A., Natto Z.S., Thompson V., Rekow D., Eckert S., Weber H.P. (2019). Ten-year survival of pressed, acid-etched e.max lithium disilicate monolithic and bilayered complete-coverage restorations: Performance and outcomes as a function of tooth position and age. J. Prosthet. Dent..

[44] Dondani J.R., Pardeshi V., Gangurde A., Shaikh A., Mahule A., Deval P. (2023). Comparative Evaluation of Wear of Natural Enamel Antagonist Against Glazed Monolithic Zirconia Crowns and Polished Monolithic Zirconia Crowns: An In Vivo Study. Int. J. Prosthodont..

[45] Almejrad L., Almansour A., Bartlett D., Austin R. (2024). CAD/CAM leucite-reinforced glass-ceramic for simulation of attrition in human enamel in vitro. Dent. Mater..

[46] Murbay S., Yeung S.K.W., Yip C.Y., Pow E.H.N. (2023). Assessing Enamel Wear of Monolithic Ceramics with Micro-CT and Intra-oral Scanner. Int. Dent. J..

[47] Krejci I., Lutz F. (1990). In-vitro test results of the evaluation of dental restoration systems. Correlation with in-vivo results. Schweiz. Monatsschr. Zahnmed..

[48] Aladağ A., Oğuz D., Çömlekoğlu M.E., Akan E. (2019). In vivo wear determination of novel CAD/CAM ceramic crowns by using 3D alignment. J. Adv. Prosthodont..

[49] Ohlmann B., Trame J.P., Dreyhaupt J., Gabbert O., Koob A., Rammelsberg P. (2008). Wear of posterior metal-free polymer crowns after 2 years. J. Oral Rehabil..

[50] Hickel R., Peschke A., Tyas M., Mjör I., Bayne S., Peters M., Hiller K.-A., Randall R., Vanherle G., Heintze S.D. (2010). FDI World Dental Federation: Clinical criteria for the evaluation of direct and indirect restorations—Update and clinical examples. Clin. Oral Investig..

[51] Suputtamongkol K., Anusavice K.J., Suchatlampong C., Sithiamnuai P., Tulapornchai C. (2008). Clinical performance and wear characteristics of veneered lithia-disilicate-based ceramic crowns. Dent. Mater..

[52] Krejci I., Lutz F., Reimer M., Heinzmann J.L. (1993). Wear of ceramic inlays, their enamel antagonists, and luting cements. J. Prosthet. Dent..

[53] Monasky G.E., Taylor D.F. (1971). Studies on the wear of porcelain, enamel, and gold. J. Prosthet. Dent..

[54] Lee A., Swain M., He L., Lyons K. (2014). Wear behavior of human enamel against lithium disilicate glass ceramic and type III gold. J. Prosthet. Dent..

[55] Zurek A.D., Alfaro M.F., Wee A.G., Yuan J.C., Barao V.A., Mathew M.T., Sukotjo C. (2019). Wear Characteristics and Volume Loss of CAD/CAM Ceramic Materials. J. Prosthodont..

[56] Krejci I., Lutz F., Reimer M. (1994). Wear of CAD/CAM ceramic inlays: Restorations, opposing cusps, and luting cements. Quintessence Int..

[57] Nawafleh N.A., Mack F., Evans J., Mackay J., Hatamleh M.M. (2013). Accuracy and reliability of methods to measure marginal adaptation of crowns and FDPs: A literature review. J. Prosthodont..

[58] Mendřický R. (2016). Determination of measurement accuracy of optical 3D scanners. MM Sci. J..

[59] Bajoghli F., Sabouhi M., Nosouhian S., Davoudi A., Behnamnia Z. (2015). Comparing the Accuracy of Three Different Impression Materials in Making Duplicate Dies. J. Int. Oral Health.

[60] Gujjarlapudi M.C., Reddy S.V., Madineni P.K., Ealla K.K., Nunna V.N., Manne S.D. (2012). Comparative evaluation of few physical properties of epoxy resin, resin-modified gypsum and conventional type IV gypsum die materials: An in vitro study. J. Contemp. Dent. Pract..

[61] Ahlholm P., Sipilä K., Vallittu P., Jakonen M., Kotiranta U. (2018). Digital Versus Conventional Impressions in Fixed Prosthodontics: A Review. J. Prosthodont..

[62] Pinto R.J., Casado S.A., Chmielewski K., Caramês J.M., Marques D.S. (2024). Accuracy of different digital acquisition methods in complete arch implant-supported prostheses: An in vitro study. J. Prosthet. Dent..

